# Exploring the determinants of mental health, wellbeing, and lifestyle in 8–11 year old children with type 1 diabetes and their healthy counterparts in Kuwait

**DOI:** 10.1371/journal.pone.0272948

**Published:** 2022-12-12

**Authors:** Afrah Alazmi, Simon Viktor, Mihela Erjavec

**Affiliations:** School of Human and Behavioural Sciences, Bangor University, Bangor, United Kindom; Flinders University, AUSTRALIA

## Abstract

Type 1 diabetes is a chronic disease with an early onset, but little is known about its psychological effects in middle childhood. The present study was the first to explore the relationship between mental health, wellbeing, and lifestyle of 8–11 years old children with Type 1 diabetes and their parents, and a healthy comparison group. A total of 200 parent-child dyads were recruited in diabetic clinics and from primary schools in Kuwait. Both groups completed a series of behavioural and physical assessments relating to health, wellbeing, and lifestyle. A significant relationship was found between higher Body Mass Index (BMI) and poorer mental health, including low academic self-esteem, depression, and anxiety, in the diabetes group. This group had significantly higher mean scores in mental health problems, and lower scores in wellbeing, compared with control group. Both groups had poor dietary habits and low levels of physical activity. Unlike previous studies, no differences were found between parents’ mental health for children with Type 1 diabetes and parents of the control group. Although elevated problem scores on a variety of indices remained within normal range, the pattern of results indicates that children with diabetes would profit from early screening and preventative intervention to reduce the likelihood of psychological and behavioural difficulties later on.

## 1 Introduction

Type 1 diabetes is an immune-associated disease caused by the destruction of islet ß cells in the pancreas, usually leading to absolute insulin deficiency [1–3]. It is a life-changing condition that involves daily glucose monitoring, insulin therapy, and carbohydrate counting [4]. Poor disease management and inadequate glycaemic control can be serious, leading to short- and long-term complications that affect children’s quality of life [5]. Even with good management, there is often an increase of body mass index (BMI) compared to children without Type 1 diabetes [6], which is especially significant in the context of increasing rates of childhood overweight and obesity worldwide. Kuwait has the largest percentage of overweight children, regardless of diabetes, in the Middle East [6, 7], and one of the largest incidence rates of Type 1 diabetes in the world [7].

A relationship between a diagnosis of Type 1 diabetes, wellbeing, and child mental health, was often reported in the existing literature [8, 9]. It is likely that a diagnosis leads to additional mental health challenges. Compared to children without this diagnosis, those affected are more likely to experience depression, anxiety, behavioural problems, and attentional difficulties [10, 11]. Children with Type 1 diabetes may also have more challenges with self-esteem compared to controls [12, 13]. A possible reason for this is that children with Type 1 diabetes are less likely to have prosocial peer support and experience more occurrences of bullying, especially those with unmanaged diabetes [14, 15]. It had also been reported that girls with Type 1 diabetes may develop a more accurate perception of their body compared to girls who do not have Type 1 diabetes [16].

Having a child with Type 1 diabetes presents many challenges for parents. The possibility and severity of long-term complications, as well as the increased responsibility of having a child with this diagnosis, may become “all-consuming” for parents [17]. These parents tend to experience significantly more parenting stress, depression, and anxiety, especially with newly diagnosed children; they also may report lower quality of life, compared to controls [18–20]. Unsurprisingly, parents without adaptive coping strategies have been reported to be more likely to experience higher subjective caregiving burden, which leads to an increase in depression severity [21]. However, parents who use positive coping strategies, especially utilising familial support, may be less likely to experience depression, or may have a reduction in its severity [22].

To this date, the majority of the studies focusing on Type 1 diabetes have been conducted in Western countries. Perhaps partly because of cultural stigma, there is limited research conducted in Middle Eastern countries [23]. Overall, the existing literature focused more on adolescents, and children in primary age have been underrepresented, even though the incidence of mental health problems in this age group had been growing for some time [24]. Most of the studies to date have looked at the relationships between the psychological variables, while lifestyle factors were seldom taken into account [24]. This is an omission, because healthy lifestyle plays a significant role in improving mental health, psychiatric, and medical diseases [25].

Our study had been designed to fill some of these gaps in the literature. Our overall aim was to explore whether primary-school aged children with Type 1 diabetes experience poorer mental health, have poorer lifestyle, and report more problems than their peers who do not have this diagnosis. To make these comparisons, we have administered the same measures to both cohorts in Kuwait. We investigated these children’s mental health indices and also examined the influence of lifestyle factors such as habitual eating behaviour and physical activity, which are important for physical and mental health, prevention of eating disorders, and general well-being across ages [26]. We investigated psychological factors such as self-esteem, coping skills, and sleep habits, as well as parental coping skills, fear, shame, mental health, and parenting skills, and their relationship to child well-being. Parental variables have been shown to be relevant in older children, and we considered that they may be of considerable importance for our younger, primary school age sample.

## 2 Methods

### 2.1 Design and sample

The study was performed in Kuwait between July and December of 2019, utilising a cross-sectional, self-report design. The study group included 100 children aged 8 to 11 years with Type 1 diabetes and their parents. The children were under follow-up care from three paediatric diabetes clinics. A matched control group included 100 children without diabetes and their parents from four schools; they were matched on gender and age. No significant differences in demographic variables was noted between the two groups (all *p*>.05). Demographic and clinical characteristics of children and their parents are shown in Tables 1 and 2, respectively.

**Table 1:** Demographic and clinical characteristics of children.

Characteristics	Diabetes cohort (*n* = 100)	Control cohort (*n* = 100)
HbA1c scores*	46 under 7.5 and 54 over 7.5	-
Therapy type	84 insulin injection and 16 pump	-
Children’s gender	54 girls and 46 boys	50 girls and 50 boys
Median age	10 years (Range 8–11 years)	10 years (Range 8–11 years)
Median weight	39 kg (Range 20–106 kg)	34 kg (Range 20–55 kg)
Median height	139 cm (Range 125–164 cm)	133 cm (Range 122–150 cm)
Median BMI percentile	82% (Range 1–99%)	85% (Range 1–98%)
Nationality	84 Kuwaiti and 16 non-Kuwaiti	96 Kuwaiti and 4 non-Kuwaiti

*Note: The International Society for Pediatric and Adolescent Diabetes (ISPAD, 2018) recommended the score of less than 7.5 for children with diabetes as healthy/desirable range. HbA1c is the standard medical measure of average blood sugar concentration over the period of 8–12 weeks [27].

**Table 2:** Demographic and clinical characteristics of parents.

Characteristics	Parents of diabetes cohort (*n* = 100)	Parents of control cohort (*n* = 100)
Parent gender	88 mothers and 12 fathers	90 mothers and 10 fathers
Parent median age bracket	35–44 years	35–44 years
Median household size	6 Members (Range 3–8)	5 Members (Range 3–8)
Parental education	3 none, 12 secondary school, 32 college, 43 bachelor’s degree, 1 master degree, 9 doctorate degree	3 secondary school, 19 college, 72 bachelor’s degree, 3 master degree, 3 doctorate degree
Parental employment status	1 home carer, 21 unemployed, 1 self-employed, 1employed part-time, 76 employed full time	1 home carer, 6 unemployed, 3 self-employed, 1 working from home, 89 employed full time
Parental history of mental health problems	8 reported history of mental health problems	4 reported history of mental health problems

### 2.2 Procedure

All study procedures were granted ethical approval by Bangor University (UK), Kuwait Ministry of Health, and Kuwait Ministry of Education.

In the paediatric clinic, children and their parents were selected by a nurse and approached during their regular visits. The nurse asked the parents whether they would be interested in participating in a research study while in the waiting room prior to their consultation with the doctor. If they agreed to participate, the nurse took them to a meeting room provided by the hospital; this ensured anonymity and privacy. The researcher provided a written consent form and an information sheet for parents to complete before participating. Parents and children were asked to complete measures related to mental health, well-being, and lifestyle. The researcher was available to assist if necessary, and to clarify or rephrase questions for the children. Height, weight, and blood glucose measurements were taken from children’s pre-existing records, from the clinic. The questionnaires took less than an hour to complete in all cases. Parent and child dyads were thanked for their participation but no incentives or gifts were offered.

For the control group, we collected the data from four schools chosen by the Ministry of Education. The parental questionnaires were sent home with the child for parents to complete, along with an information sheet and consent form. The children completed their questionnaires at school in their class. The researcher was present, read the questions aloud, and assisted individual children if necessary. Children’s weights and heights, recently measured by the school nurse, were taken from pre-existing records.

### 2.3 Measures

Measures were chosen for their suitability for primary school aged children, widespread use in previous research, and because they were validated and/or showed good internal consistency. They were translated from English to Arabic except for the Coppersmith Self-Esteem Inventory-School Form, The Child Behavior Checklist, The World Health Organization Five Wellbeing Index, and the Strengths and Difficulties Questionnaire, which were already available and validated in Arabic. The measures were translated by a professional from English to Arabic and back to English again; this forward- and back-translation procedure provided an accurate translation of the measures [28]. All the Arabic scripts were also checked by the researcher who is a native Arabic speaker.

**Fig 1:**
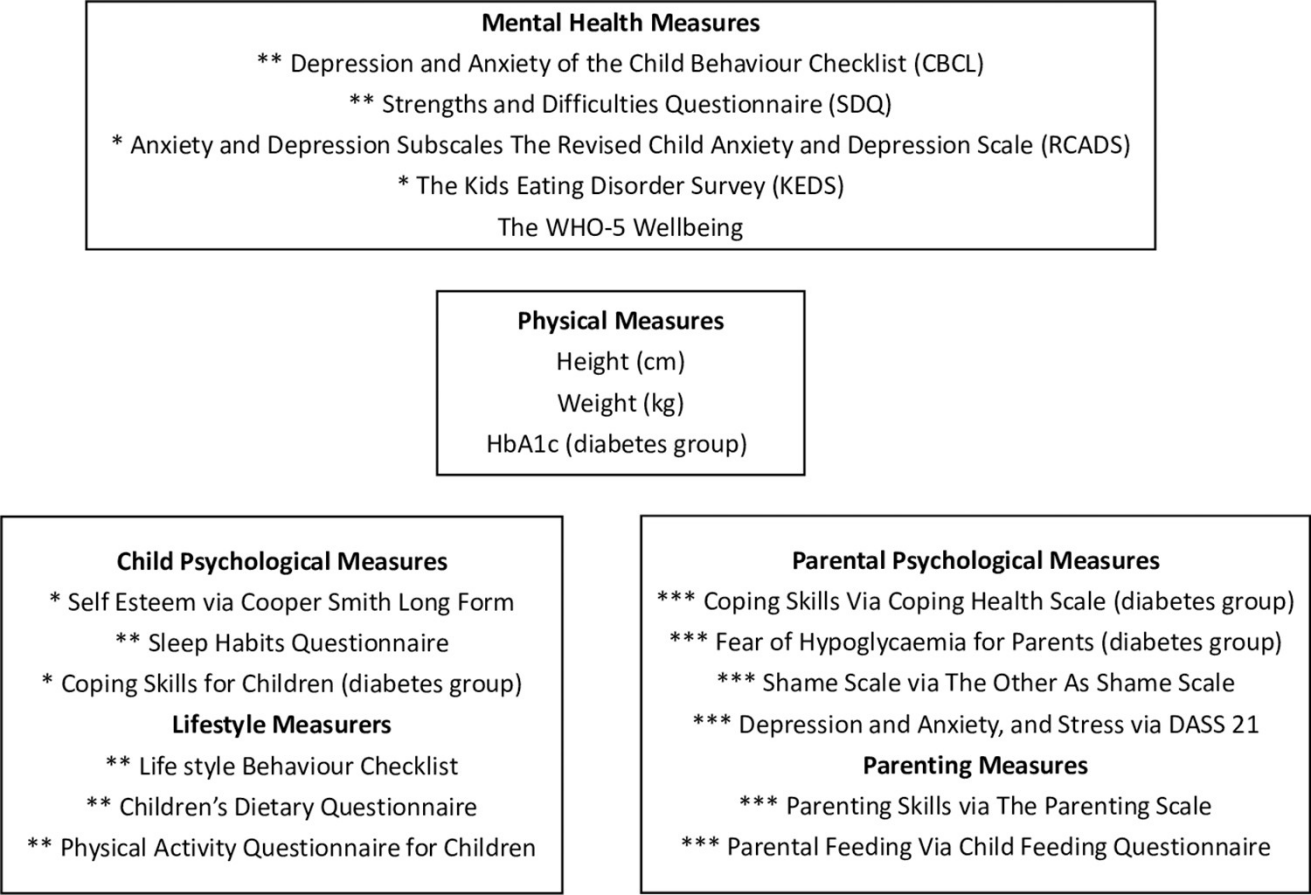
Measures used in the study. *Note*:* measures completed by children, ** child measures completed by parents, *** measures completed by parents. The WHO-5 Wellbeing measure was completed by both parents and children.

Fig 1 shows the measures used in the present study. We have administered a battery of questionnaires to parents, asking about their own mental health and parenting, and also about their children’s mental health, wellbeing, and lifestyle (including sleep quality, dietary habits, and physical activity). We have also asked children to provide answers about their own mental health (including anxiety, depression, and disordered eating), self-esteem, and coping skills.

### 2.4 Supporting information

Supporting information file contains (i) full descriptions of each measure, subscales, and scoring; (ii) Chronbach alphas obtained for each measure; and (iii) descriptive statistics for all measures.

## 3 Results

### 3.1 Preliminary analysis and decision rules

Exploratory data analysis techniques were performed to identify the range, mean, and standard deviation of Child Self-Completed Measures, Parent-Completed Child Measures, and Parent-Completed Self-Report Measures (S2 Table in S1 File). Variables that were found to score higher than ±2 for skew and kurtosis were investigated with non-parametric tests [29], because they did not meet the requirements of normality, linearity, or parametric assumptions. When parametric assumptions were met, one-way ANOVA, independent samples *t*-tests, Pearson’s *r* product moment correlations, and hierarchical (or linear) regressions were employed. When parametric assumptions were violated, Mann-Whitney U and Spearman’s Rho tests were used.

Chi-squares were performed with crosstabs to identify any differences in the parent and child demographic variables for the diabetes and control group. None were identified, meaning that the samples for each group were well matched. The additional findings related to diabetes management (insulin injection or insulin pump) and BMI classifications (e.g., overweight and obese) are presented in the appropriate section.

Each set of inferential tests also included the corresponding effect size calculation when necessary (e.g., Cohen’s *d*, Cohen’s *f*-squared, eta-squared, and post-hoc power analysis). The raw scores for the Child Behaviour Check List subscales were analysed instead of the *T-*scored data in accordance with Pandolfi, Magyar, and Dill [30] and Holmes et al. [31], who established no differences in the findings reported from using the raw scores in the analysis as opposed to the *T*-scores. In addition, the *T*-scored data for the RCADS was also analysed in this study [32].

The analyses were exploratory in nature but we have considered indices of mental health as outcome variables where appropriate. Chronbach alphas obtained for each measure in the present study and descriptive statistics for all measures are presented in S1 File. There were no missing data for any of the participants. The findings are presented in three sections for: (i) diabetes group; (ii) control group; and (iii) comparisons between the scores for the diabetes and control group. Only statistically significant results are listed in each section.

### 3.2 Diabetes group

Independent samples *t*-tests were carried out to identify differences between blood test scores (HbA1c) when comparing children with managed vs. unmanaged diabetes according to The International Society for Paediatric and Adolescent Diabetes (ISPAD, 2018) criteria. The managed group (*n* = 46) had an HbA1c of less than 7.5%, and the unmanaged group (*n* = 54) had an HbA1c of 7.5% or more. A significant difference was found between the groups when comparing their scores for parental shame; the parents in the unmanaged group (*M* = 58.48, *SD* = 8.84) reported being more manipulative than those in the managed group (*M* = 54.52, *SD* = 9.63), *t*(98) = -2.14, *p* < .05, *d* = 0.40 (small-medium effect size).

A Mann-Whitney U test found that the managed group (Median = 5.00) consumed more water than the unmanaged group (Median = 4.00), *U* = 910, *z* = -2.53, *p* = .01.

#### BMI percentiles for diabetes group

A Pearson’s *r* correlation was conducted to identify the relations between the children’s Body Mass Index (BMI) percentiles and study measures. As shown in Table 3, a number of significant positive correlations were found to exist between the scores for BMI percentile; eating disorder survey body dissatisfaction, *T*-scored RCADS panic disorder, *T*-scored RCADS depression, and *T-*scored RCADS general anxiety scores. A negative correlation was also found to exist between BMI percentile and eating disorder scores items 1–7, parental DASS-21 stress scores, and children’s self-esteem academic score.

**Table 3:** Pearson’s correlations for diabetes group bmi percentile with other study variables.

Measure	2	3	4	5	6	7	8
1 BMI Percentile	-.315**	.322**	.236*	.202*	.268*	-.226*	-.213*
2 Eating disorder survey items 1–7		-.128	-.171	-.001	-.074	.010	.091
3 Eating disorder survey body dissatisfaction			.267**	-.038	.310**	-.062	-.136
4 *T*-scored RCADS panic disorder				-.067	.478**	-.224*	-.272**
5 *T*-scored RCADS depression					-.040	-.165	-.204*
6 *T*-scored RCADS generalised anxiety						-.146	-.265**
7 DASS-21 stress							.224*
8 Self-esteem academic							

**Correlation is significant at the .001 level (two-tailed)

*Correlation is significant at the .05 level (two-tailed)

#### BMI classifications for diabetes group

The BMI scores for the diabetes group were classified into four main categories: underweight; healthy weight, overweight, and obese. The underweight group was excluded from the analysis (*n* = 1). Children whose BMI was classified as obese scored higher on eating disorders survey body dissatisfaction; and *T*-scored RCADS panic disorder, general anxiety, and sleep awake earlier. Overweight children scored higher on *T*-scored RCADS obsessive compulsive. Interestingly, children whose BMI was classified as healthy weight were found to be scoring higher on the eating disorder survey items 1–7 than those who were either overweight or obese (see Table 4).

**Table 4:** Diabetes group one-way ANOVA for BMI classification.

Key	Healthy weight (*n* = 54) Mean (Sd)	Overweight (*n* = 24) Mean (Sd)	Obese (*n* = 21) Mean (Sd)	*F*	*p*	*η^2^*
1	5.18 (2.53)	3.83 (2.66)	3.24 (2.07)	5.63	.005	0.10
2	0.98 (1.02)	1.42 (1.28)	2.43 (1.03)	11.18	< .001	0.19
3	54.37 (8.04)	56.38 (8.50)	61.38 (9.56)	5.16	.007	0.10
4	42.89 (6.33)	45.56 (9.33)	47.48 (6.83)	3.30	.041	0.06
5	45.93 (9.70)	51.83 (8.11)	51.81 (9.05)	5.03	.008	0.09
6	7.93 (2.60)	8.43 (2.99)	6.33 (3.23)	3.46	.036	0.07

*Key*: 1 = Eating disorders survey items 1–7; 2 = Eating disorders survey body dissatisfaction, 3 = *T*-scored RCADS Panic disorder, 4 = *T*-scored RCADS General anxiety, 5 = *T*-scored RCADS Obsessive compulsive, and 6 = Sleep: Morning wake-up. Eta-squared values: 0.01 = small effect; 0.06 = medium effect, and 0.14 or higher = large effect.

#### Mental health and wellbeing variables for the diabetes group

A Pearson’s *r* bivariate correlation was conducted to identify the relations between children’s mental health and wellbeing scores (see Table 5). A number of significant negative correlations were found to exist between the child’s wellbeing and *T-*scored RCADS social phobia, *T*-scored RCADS depression, *T*-scored RCADS generalised anxiety, and eating disorder survey body dissatisfaction scores. This demonstrates that the elevated scores for (non-clinical) measures of social phobia, depression, generalised anxiety, and eating disorder survey body dissatisfaction may be associated with poorer wellbeing in children with diabetes. A positive correlation was also found to exist between the parents’ wellbeing and children’s self-esteem school academic scores.

**Table 5:** Pearson’s correlations for mental health and wellbeing variables for diabetes group.

Measure	2	3	4	5	6	7	8	9	10	11	12	13	14	15	16	17	18
1 Child welling	-.215*	-.248*	-.217*	-.264*													
2 *T*-scored RCADS social phobia						-.216*				-.197*							
3 *T*-scored RCADS depression											-.247*						
4 *T*-scored RCADS generalized anxiety																	
5 Eating body dissatisfaction																	
6 Parental welling						.305*											
7 Self-esteem academic																	
8 Binge eating								-.222*	-.225*	.258*							
9 Coping wishful thinking																	
10 Sleep morning																	
11 Coping avoidance																	
12 Self-esteem total																	
13 Raw CBCL withdrawn depression													.231*	.217*			
14 Parental shame																	
15 Parental feeding responsibility																	
16 Raw CBCL anxiety/depressed																.231*	-.232*
17 Coping emotional reaction																	
18 Coping subscale 3																	

**Correlation is significant at the .001 level (two-tailed)

*Correlation is significant at the .05 level (two-tailed).

Eating disorder survey binge eating scores were inversely correlated with coping avoidance, coping wishful thinking, and sleep morning wake up scores, indicating that children with poorer coping and sleep-related problems had elevated disordered eating behaviour.

Social phobia scores showed a significant negative correlation with coping avoidance, and academic self-esteem scores; children with an elevated social phobia appeared to engage in fewer avoidance techniques and had lower academic self-esteem. A significant inverse relation between separation anxiety and self-esteem total scores was also observed.

Higher scores for the raw CBCL withdrawn depressed subscale showed significant positive relation with parental shame, and with parents feeding perceived responsibility scores. There were also significant inverse relations between the raw CBCL anxiety/depressed and coping emotional reaction scores, and coping subscale 3 score.

#### The effects of diabetic management type

The scores for the main variables were inspected by diabetes management type to identify any differences on the main variables. Children whose diabetes was managed with a insulin pump scored higher on self-esteem general and social than those whose diabetes was managed by insulin injection. Children whose diabetes was managed by insulin injection scored higher on eating disorder survey binge eating and *T*-scored RCADS separation anxiety. The parents of the children who were managed by insulin pump registered more HFS behaviour scale related problems than those parents whose child was managed by insulin injection (see Table 6). It is worth noting that the children with diabetes in this study were primarily managed by insulin injection.

**Table 6:** Diabetes management differences on main variables.

Variable	Injections (*n* = 84) Mean (Sd)	Pump (*n* = 16) Mean (Sd)	*t*	*p*	*d*
Self-esteem general	11.86 (3.28)	14.00 (2.56)	-2.47	.015	0.73
Self-esteem social	3.27 (1.70)	4.25 (1.65)	-2.11	.037	0.59
Binge eating score	0.53 (0.83)	0.06 (0.25)	*4.31	< .001	0.77
*T*-scored RCADS Separation anxiety	56.32 (8.20)	51.56 (7.81)	4.76	.035	0.59
HFS Behaviour scale	3.76 (0.81)	4.21 (0.52)	-2.15	.034	0.66

* Adjusted *t-* and *p*-value reported because Levene’s homogeneity of variance test was violated. Cohen’s *d* values: 0.20 = small effect; 0.50 = medium effect, and 0.80 = large effect (Cohen, 1988).

#### Regression analysis for diabetes group

Regression analysis were run to establish further the relations between the predictors and child mental health outcome variables after controlling for the effect of diabetes management type. Tolerance and Variance Inflation Factors (VIF) were examined to identify any collinearity issues in the models. This is important as it means the independent variables do not influence one another too much. Therefore, it can be identified to what extent each independent variable influences the dependent variables, separately. Tolerance varies between 0 and 1.00, for example, when the value is greater than 1.00 it means that the variable is completely uncorrelated with other independent variables. Moreover, the VIF value is supposed to be less than 2.00 [29].

The model for predicting children’s eating disorder survey binge eating scores accounted for 18.9% of the unique variance *F*(4,95) = 5.54, *p* < .001, *f^2^ =* 0.23 (small effect size), power = .98. The model for predicting children’s separation anxiety scores accounted for 17.5% of the unique variance *F*(3, 96) = 6.77, *p* < .001, *f^2^* = 0.21 (small effect size), power = .98. The regression diagnostic tests applied to each model show that no multicollinearity issues occurred: model 1 VIF = 1.01 to 1.09 and Tolerance .92 to .99 and for model 2: VIF = 1.00 to 1.04 and Tolerance .96 to 1.00. For both models, *β* values and *p* values are shown in Table 7.

**Table 7:** Regression findings for diabetes group.

Outcome Variables	Predictor Variables	*β*	*p value*
1 Eating disorder survey binge eating	Diabetes management type	-.222	.026
	Coping avoidance	-.284	.003
	Coping wishful thinking	-.211	.025
	Sleep morning wake up	-.124	.201
2 *T*-scored *RCADS* Separation Anxiety	Diabetes management type	-.212	.035
	Self-esteem total score	-.215	.032
	Sleep bed time	.293	.002

### 3.3 Control group

#### BMI percentile control group

A Pearson’s *r* correlation was conducted to identify the relations between the BMI percentiles and the main study variables scores. As shown in Table 8, all eating disorder survey subscale scores were found to correlate with BMI percentile: items 1–7 score, binge eating score, and body dissatisfaction score. In other words, higher weight status was associated with higher disordered eating indices. However, a negative correlation was found between BMI percentile and peer problem scores from the strengths and difficulties questionnaire (SDQ).

**Table 8:** Pearson’s correlations for control group BMI percentile.

Measure	2	3	4	5
1 BMI Percentile	.324**	.360**	.203*	-.204**
2 Eating disorder survey items 1–7		-.499**	-.664**	-.111
3 Eating disorder survey binge eating			.568**	-.094
4 Eating disorder survey body dissatisfaction				-.050
5 SDQ Peer Problem				-

**Correlation is significant at the .001 level (two-tailed)

*Correlation is significant at the .05 level (two-tailed)

A Spearman’s Rho correlation was conducted on the scores for BMI percentiles and lifestyle variables scores. As shown in Table 9, a significant negative correlation was found between BMI percentiles and sleep waking during the night, and positive correlations with the amount and frequency of physical activity at the weekend. Surprisingly, higher weight status was associated with less interrupted sleep and more activity in the control group.

**Table 9:** Spearman’s Rho correlations for control group BMI percentile.

Measure	2	3	4
1 BMI Percentile	-.284**	.296**	.291**
2 Waking during night		-.041	-.086
3 Physical activity weekend total			.088
4 Physical activity weekend frequency			-

**Correlation is significant at the .001 level (two-tailed)

*Correlation is significant at the .05 level (two-tailed).

#### BMI classifications for control group

In a similar manner to the diabetes group, the BMI scores for the control group were reclassified into four categories; the underweight group was excluded from the analysis (*n* = 3). The healthy weight children scored higher on eating disorder survey items 1–7 than the other BMI classifications; obese children scored higher on eating disorder survey body dissatisfaction. Both these finding are in keeping with the diabetes group. A noticeable difference to the diabetes group is that the obese children in the control group were also scoring higher on eating disorder survey binge eating, *T*-scored RCADS major depression, and lower for raw CBCL anxiety problem. Surprisingly, the healthy weight children in the control group were found to be scoring the highest on CBCL anxiety problem and sleep: waking during the night (see Table 10).

**Table 10:** Control group one-way ANOVA for BMI classification on main variables.

Key	Healthy weight (*n* = 45) Mean (Sd)	Overweight (*n* = 36) Mean (Sd)	Obese (*n* = 16) Mean (Sd)	*F*	*p*	*η^2^*
1	6.40 (1.42)	5.14 (2.17)	5.56 (1.97)	10.00	< .001	0.17
2	0.98 (1.16)	1.58 (1.40)	2.87 (1.67)	11.85	< .001	0.20
3	0.40 (0.72)	0.94 (1.16)	1.56 (1.21)	9.58	< .001	0.17
4	41.27 (5.95)	41.58 (7.54)	46.12 (5.78)	3.47	.035	0.07
5	3.24 (2.28)	2.36 (1.77)	1.44 (1.59)	5.28	.007	0.10
6	2.24 (1.48)	1.61 (1.20)	1.31 (1.30)	3.73	.028	0.07

*Key*: 1 = Eating disorders survey items 1–7; 2 = Eating disorders survey body dissatisfaction, 3 = Eating disorders survey binge eating, 4 = *T*-scored RCADS Major depression, 5 = Raw CBCL Anxiety problem, and 6 = Sleep: Waking during the night. Eta-squared values: 0.01 = small effect; 0.06 = medium effect, and 0.14 or higher = large effect.

#### Mental health and wellbeing variables in the control group

A Pearson *r* bivariate correlation was conducted to investigate the relations between mental health and wellbeing scores (see Table 11). A negative correlation was found to exist between wellbeing and *T*-scored RCADS depression scores. Children’s higher *T*-scored RCADS obsessive-compulsive scores correlated with higher parental shame and parental child weight. However, raw CBCL anxious depressed subscale scores were found to share a significant negative correlation with shame and bedtime scores.

**Table 11:** Pearson’s correlations for control group mental health and wellbeing variables.

Measure	2	3	4	5	6	7	8	9	10	11	12	13
1 Child welling	-.245*											
2 *T*-scored RCADS depression												
3 Eating survey binge eating			.568**									
4 Eating survey body dissatisfaction												
5 Eating survey items 1–7		-.499**	-.664**		.218*	.226*	.208*					
6 Self-esteem total												
7 Self-esteem academic												
8 Self-esteem general												
9 *T*-scored RCADS obsessive compulsive									.283*	.317**		
10 Parental shame												
11 Parental child weight												
12 Raw CBCL anxiety/depressed									-.197*			-.202*
13 Sleep bedtime												

**Correlation is significant at the .001 level (two-tailed)

*Correlation is significant at the .05 level (two-tailed).

Unsurprisingly, children who reported higher scores for eating disorder survey binge eating also reported higher body dissatisfaction. However, eating disorder survey items 1–7 scores had a significant negative correlation with body dissatisfaction scores and binge eating scores. Unexpectedly, eating disorder survey items 1–7 scores were positively correlated with total self-esteem, academic self-esteem, and general self-esteem scores.

### 3.4 Comparisons between the diabetes and control group

Comparison data analyses were carried out with 100 children with Type 1 diabetes, 100 control children, and their parents. Table 12 shows the differences in the variable scores by group.

**Table 12:** Diabetes and control group differences on main study variables.

Variables	Diabetes group *M (SD)*	Control group *M (SD)*	*U*	*t*-test	*p*	*d*
**Child**						
Self-esteem						
General self	12.20 (3.26)	13.55 (3.64)		2.76	.006	0.39
Social self	3.43 (1.72)	4.01 (1.47)		2.56**	.011	0.36
Home parents	3.08 (1.43)	3.91 (1.63)		3.81	< .001	0.54
School academic	3.21 (1.59)	4.16 (1.62)		4.17	< .001	0.59
Total score	10.96 (2.83)	12.81 (3.19)		4.38	< .001	0.61
Eating Disorder Survey						
Eating disorder items 1–7	4.47 (2.59)	5.58 (1.97)		3.41	.001	0.48
Binge eating	0.46 (0.78)	0.77 (1.02)		13.28	.001	1.88
Child wellbeing	17.59 (4.54)	21.15 (3.53)		6.18	< .001	0.87
RCADS						
*T*-scored Social phobia	42.30 (7.05)	35.61 (5.40)		7.53	< .001**	1.06
*T*-scored Panic disorder	56.25 (8.84) Median 56	45.56 (7.20) Median 45	46.12*		< .001*	1.32
*T*-scored Major depression	52.65 (9.34)	42.19 (6.66)		-9.11	< .001**	1.29
*T*-scored Separation anxiety	55.56 (8.28)	44.89 (6.09)		-10.37	< .001**	1.47
*T*-scored Generalised anxiety	44.38 (7.46)	36.71 (5.40)		-8.32	< .001**	1.18
*T*-scored Obsessive compulsive	48.72 (9.59) Median 49	38.07 (6.43) Median 37	78.2*		< .001*	1.30
Sleep Habits						
Bedtime	14.62 (4.16)	17.60 (6.90)		3.70	< .001**	0.52
Waking during night	3.77 (1.36) Median 4	1.88 (1.38) Median 2	61.71*		< .001*	1.37
Morning wake up	7.64 (2.99)	2.84 (2.78)		11.74	< .001**	1.66
CBCL						
Raw Depressive problems	5.15 (2.98) Median 5	3.02 (2.22) Median 3	25.24*		< .001*	0.80
Raw Anxiety problems	4.21 (1.96)	2.65 (2.08)		-5.44	< .001	0.77
Raw Anxious/depressed	5.88 (2.66)	3.78 (2.85)		5.37	< .001	0.76
Raw Withdrawn/depressed	3.41 (2.53)	1.61 (1.54)		-6.06	< .001	0.86
SDQ						
Emotional symptoms	2.73 (1.72)	1.77 (1.39)		-4.33	< .001**	0.61
Hyperactivity	2.11 (1.41)	5.28 (1.37)		16.1	< .001	2.28
Difficulties global score	11.14 (3.57)	13.48 (2.94)		5.06	< .001	0.72
Prosocial global score	3.68 (2.17) Median 3.5	2.79 (1.77) Median 2	9.27*		< .002*	0.44
**Parents**						
Parental feeding perceived responsibility	4.05 (0.86) Median 4	3.71 (0.83) Median 4	7.66*		.006	0.40
Parental feeding monitoring	3.71 (0.84)	3.46 (0.74)		-2.19	.029	0.31
Parental style sum	4.28 (0.67) Median 4	4.80 (1.12) Median 5	12.52*		< .001	0.56
Parental style laxness	4.34 (0.78)	4.60 (0.87)		2.17	.031	0.31
**Lifestyle**						
Lifestyle behaviour checklist						
Food	20.98 (8.26) Median 20	15.53 (8.21) Median 14	18.09*		< .001*	0.66
Children dietary questionnaire						
Fruits eaten in the last 7 days	6.59 (3.86) Median 6	4.83 (2.18) Median 5	19.00*		< .001*	0.56
Fruit last week	2.97 (1.41) Median 3	2.05 (1.29) Median 2	32.08*		< .001*	0.68
Vegetable in evening meal (24 hours)	1.100 (0.88) Median 1	1.21 (1.23) Median 1	4.92*		.026*	0.19
Vegetables last week	2.34 (1.43) Median 2	1.87 (1.25) Median 2	6.16*		.013*	0.34
Non-core foods past 7 days	22.43 (8.51) Median 21	27.46 (12.52) Median 25	4.50*		.034	0.46
Variables	Diabetes group *M (SD)*	Control group *M (SD)*	*U*	*t*-test	*P*	*d*
Sweetened beverage last 24 hours	1.07 (1.04) Median 1	1.99 (1.35) Median 2	15.07*		< .001*	0.57
Fruits eaten average daily portion	1.66 (0.72) Median 1.5	1.32 (0.56) Median 1.1	19.73*		< .001*	0.52
Non-core foods average daily portion	3.20 (1.21) Median 3	3.92 (1.78) Median 3.6	4.50*		.034*	0.47
C-PAQ						
Frequency on weekdays	36.71(35.08) Median .00	2.67 (2.26) Median .00	10.73*		.001*	0.28
Frequency on weekends	0.00 (.0.00) Median .00	0.23 (0.69) Median .00	10.73*		< .001**	0.00*

* Non-parametric analysis used due to the data not meeting the requirements of normality

** Adjusted *df* and *t*-value reported because Levene’s homogeneity of variance test was violated.

#### Differences for diabetes and control group by BMI classification

A one-way ANOVA with six groups was used to identify how children classified by their BMI as healthy weight, overweight, and obese in the diabetes and control group were scoring compared to each other. We examined the eating disorders survey items 1–7, binge eating, and body dissatisfaction, because both groups were found to be showing differences on these variables by BMI classification. ANOVA showed that children with a healthy weight classification in the control group scored the highest on the eating disorder survey items 1–7 compared to the other BMI classifications in either the diabetes or control group, *F*(5,190) = 8.37, *p* < .001, *η^2^* = 0.18 (large effect).

For eating survey binge eating scores, those in the control group classified as obese scored higher than the other BMI classifications in either the diabetes or control group, *F*(5,190) = 6.69, *p* < .001, *η^2^* = 0.15 (large effect). They also scored higher for body dissatisfaction, *F*(5,190) = 9.35, *p* < .001, *η^2^* = 0.20 (large effect). No other differences on main variables were identified (*p*>.05).

#### Parent variables

Parents in the control group had significantly lower mean scores for feeding responsibility and had less monitoring over the feeding of their children. Parents of children with Type 1 diabetes had significantly lower mean scores on parenting sum, parenting laxness, and parenting verbosity than parents in the control group. However, there were no significant differences between the parents of each group on their reported levels of external shame, mental health, and wellbeing (*p*>.05).

#### Child variables

Children with Type 1 diabetes had significantly lower mean scores for general self-esteem, social, home parents, school academic, and total self-esteem than the control group. There were no differences between the two groups on eating disorder survey body dissatisfaction. Children with Type 1 diabetes had significantly lower mean scores for the eating disorder survey items 1–7, while the control had significantly lower mean scores for eating disorder survey binge eating.

The state of wellbeing in the children with Type 1 diabetes was less positive than that of the control group. Compared with the control group, the children with Type 1 diabetes had significantly higher mean scores of *T*-scored RCADS subscales, raw CBCL subscales, and SDQ emotional symptoms subscale. Significantly lower mean scores in sleep habits were observed in children with Type 1 diabetes group, while the control group had significantly lower mean scores of sleep waking during night and sleep morning wake up.

#### Lifestyle variables

The control group had significantly lower median scores for lifestyle food, fruits in the past 7 days, in the last week, and the average daily portions in their diet compared to children with Type 1 diabetes. However, children with Type 1 diabetes reported significantly lower median scores of vegetable meals in the last 24 hours, less non-core food for the past 7 days, less sweetened beverages in 24 hours, and a daily portion of non-core foods, compared to the control group. Children with Type 1 diabetes had significantly higher median scores than the control group when comparing the frequency of physical activities. However, it should be noted that, in both cohorts, diet was relatively poor and levels of activity low (see Table 12 and S1 File).

## 4 Discussion

The present study was the first to investigate mental health, wellbeing, and lifestyle factors in young children with Type 1 diabetes and their healthy counterparts in Kuwait. The findings demonstrate the connections between a range of lifestyle and self-evaluative variables such as eating habits, self-esteem, shame, and peer interactions, with children’s mental health and wellbeing. In most part, our results align with the findings reported in the existing literature from samples with broader age range, older children, and those from different cultures. However, some of the results were surprising.

### Main findings for the diabetes group

The main findings for the diabetes group analysis showed that some differences were due to HbA1c grouping (managed or unmanaged); BMI percentile and classification (e.g., obese or overweight), and diabetes management type (insulin injection or pump). For HbA1c, the only notable findings were for the unmanaged group, where parents reported an increase in manipulativeness and the children consumed less water than the managed group.

More findings were associated with BMI percentile and classification; three positive correlates were found to exist between *T*-scored RCADS mental health and BMI percentile scores. BMI percentile scores also shared one negative correlate (items 1–7) and one positive correlate (body dissatisfaction) with the eating disorder survey scores. These correlational findings are further supported by the one-way ANOVAs. Obese children were found to be scoring higher on body dissatisfaction and the panic and anxiety indices of the RCADS. Those who were overweight scored higher on the RCADS obsessive compulsive index. This pattern of correlates and ANOVA analyses clearly demonstrate the relations between BMI, mental health, and disordered eating patterns in the diabetes group. Other notable findings include inverse relations between BMI percentile and parental stress and children’s self-esteem academic. We also found that healthy weight children scored higher on items 1–7 of the eating disorders survey and that obese children were waking earlier than the other BMI classifications.

Differences on self-esteem, mental health, and disordered eating indices were observed when investigating the role of diabetes management type. Children who were managed by insulin injection (*n* = 84) were found to score lower on self-esteem general and social and higher on *T*-scored RCADS separation anxiety and eating disorder survey binge eating. Parents of children managed by insulin pump (*n* = 16) recorded higher scores for the HFS behaviour scale than those managed by insulin injection, implying that they may be engaging in more avoidance behaviour to reduce their child’s hypoglycaemic risk [33].

In summary, the elevated scores on the RCADS mental health and eating disorder survey items within the diabetes group were associated with differences in BMI percentile, BMI classification, diabetes management type, and with poorer self-esteem, coping behaviour, and sleep-related problems. This conclusion is supported by the regression analyses that show both binge eating and *T*-scored RCADS separation anxiety are predicted by a combination of being maintained by insulin pump injection, self-esteem, coping behaviour, and sleep-related problems. Broadly, our findings correspond to the existing literature: Melnyk et al. [34] and Halfon et al. [35] reported a correlation between scores for depression, low self-esteem, school problems, number of missed school days, and a high BMI. A negative relationship found to exist with binge eating, coping avoidance, and sleep habits in our study is consistent with the findings reported by Burt et al. [36]. Poor wellbeing in children with diabetes may be associated with depression, general anxiety, social phobia, and body dissatisfaction. A similar association was also reported by de Wit et al. [9], who found that children with Type 1 diabetes reported higher social phobia scores and lower academic self-esteem and avoidance technique scores; whereas those with higher levels of anxiety reported low self-esteem. These finding are also in keeping with those reported by Ayla et al. [37] and Yemane et al. [38].

In our sample, the scores for mental health and disordered eating indices that were elevated within the diabetes group did not yet fall into a clinical range. Nevertheless, our findings imply that the screening and assessment of younger children with Type 1 diabetes may be needed to identify those who may profit from early (preventative) intervention.

### Main findings for the control group

Our study was the first to explore the relationship between lifestyle, wellbeing, and mental health indices of healthy primary-school age children from an Arab country.

The BMI percentiles for the healthy control group shared positive correlations with three disordered eating indices (eating disorder survey items 1–7; binge eating and body dissatisfaction), as previously reported in the literature by Munkholm et al. [39]. These findings differ to those for the diabetes group, which showed an inverse relation between BMI percentile and eating disorder survey items survey 1–7. An additional difference observed is the inverse relation between the strengths and difficulties questionnaire (SDQ) peer problems scores and BMI percentile for the control group, whereas no relations between SDQ subscale scores and BMI percentile were observed in the diabetes group.

An unexpected finding was the positive relations between BMI percentile and the amount and frequency of physical activity in the control group. This may be due to the number of overweight and obese children in each group (control *n* = 52 and diabetes *n* = 45), or because the scores for physical activity were lower than expected for this age-range in each group. This finding needs to be replicated in another study to further elucidate the relations between BMI and physical activity.

The main findings in relation to BMI classification for the control group are the higher scores for obese children on eating disorder survey binge eating and *T*-scored RCADS major depression. They may be at risk of engaging in emotional or loss of control eating to regulate depression related symptoms [40].

In general, the pattern of BMI classification for the controls is identical to that for the diabetes group when it comes to eating disorder survey items 1–7 and body dissatisfaction. An unexpected finding is the scores for healthy weight children on the raw CBCL anxiety problem scale and sleep waking during the night. This was contrary to the findings reported by Kanellopoulou et al. [41] who found that poor sleep patterns and sleep duration are associated with higher weight status. Although these scores are elevated in our sample, they do not fall within a clinical range. The same also holds for the healthy weight children’s positive pattern of correlates for disordered eating (items 1–7) and self-esteem indices (total, academic and general) and the negative relations between wellbeing and *T*-scored RCADS depression scale.

The present study also identified the links between child mental health and other variables such as parental shame, behavioural difficulties in children, their sleep habits, and self-esteem. As was expected, better wellbeing was found to be related to fewer behavioural and emotional problems, including depressive symptoms [42]. By contrast, differences were observed between previous studies and the current study, as children with higher scores for disordered eating reported higher scores for self-esteem [43].

### Comparisons between diabetes and control group

We found that the control group scored higher on all the disordered eating variables (e.g., binge eating) than the diabetes group, Troncone et al. [44] suggested that it is most likely the result of the increased attention that children with Type 1 diabetes are forced to pay to their bodies, both in terms of function and size (weight loss/gain), and the knowledge of the value of nutrition, exercise may exacerbate a person’s self-consciousness, irrespective of BMI classification.

In our sample, diabetes group scored lower on measures of: self-esteem; eating disorder survey items 1–7, wellbeing, *T*-scored RCADS subscales (all), raw CBCL subscales (most), and sleep habits. Lower levels of self-esteem in the diabetes cohort could be linked to how a young person sees their own efficacy in the home, at school, and in other situations [41, 45]. Their higher mental health problem scores may put them at greater risk of experiencing depression, anxiety, and social phobia related problems in the future. Our findings are in line with other published studies: they could be associated with patient frustration with the differences between themselves and other children, the need to take daily insulin shots, lifestyle changes as a result of long-term disease management, and poor understanding of their condition among parents [11, 46]. Increased family conflict and low self-esteem are also likely to be linked to poor wellbeing [9]. Children with Type 1 diabetes have been reported to have more sleep disturbances, such as night-time waking, compared to their healthy counterparts, due to hypoglycaemia or parents’ night-time caregiving practices [46, 47].

Surprisingly, some indices showed that fruit and vegetable consumption was higher in the diabetes group and they consumed less non-core food (e.g., snacks), less sweetened beverages, and had higher physical activity levels than the control group. This would be good news, because healthy pattern makes blood sugar easier to control and could prevent obesity and any long-term related complications, such as cardiovascular disease and stroke. Excessive weight has been found to enhance the body’s resistance to insulin, resulting in increased insulin needs and more weight gain [26]. Unfortunately, it needs to be noted that most children’s consumption of healthful foods was extremely low in both groups, and their Median BMI was high. The same can be said about their levels of physical activity. There are probably cultural reasons for this pattern. With respect to the diabetes group, it had previously been reported that diabetic teenagers tend to avoid physical activity due to fear of hypoglycaemia [48], even though physical activity can lower HbA1C levels and improve quality of life [49, 50]. Overall, we consider that healthy lifestyle interventions promoting fruit and vegetable consumption and physical activity would benefit both cohorts.

Some variations in parental behaviours were also observed. Parents in the control group scored lower on the child feeding related variables (e.g., feeding responsibility) than the parents of children with Type 1 diabetes; this may be due to added responsibilities in the latter group related to diabetes management. The opposite trend was seen for parenting related problems (e.g., laxness), with parents of children with Type 1 diabetes scoring lower. Many previous studies have shown that parents of children with diabetes show symptoms of anxiety, depression, and stress compared to control groups [18, 19]. However, our study did not find any differences in the mental health and wellbeing scores for parents of young children with Type 1 diabetes and the control group. This could be the result of the higher sensitivity to moral values, and the higher levels of prosocial behaviours exhibited among Kuwaiti parents, as Kuwait is considered a religious country [51]. It may be that relative wealth and traditional extended family structure act as protective factors for these parents. However, there is also a possibility that parents did not want to disclose any mental health issues, as indicated by limited research regarding mental health in the Middle East, as well as the social and cultural stigma surrounding this topic [23, 52]. Almazeedi and Alsuwaidan [53] claim that stigma makes people less likely to disclose negative behaviours related to poor mental health or wellbeing. Therefore, they are less likely to seek treatment or more information regarding these issues. In addition, religion, shame on the family, and a lack of community support can also act as barriers to mental health disclosure and support in countries such as Kuwait [53].

### Strengths, limitations, and recommendations

The strengths of our research include: examining the relations between mental health, wellbeing, and lifestyle factors (previously not used in a single sample) in diverse cohorts that reflect the population but were nevertheless well matched at the outset on a range of demographic variables; recruiting 8–11 year old primary-aged children (rather than older children or a very broad range of ages, as was typical in the existing literature); looking at parent-child dyads; and reporting the result from an under-researched population.

We also note some limitations to the conclusions that can be drawn from the study. This research relied mainly on self-report measures, which can be prone to biases in both children and adults [35]; however, the alternatives would have been both impractical and, in some cases, arguably less reliable. We asked the children to complete some of the questionnaires; similar data collection methods to ours have often been used in the existing literature with this age group and measures [54–56]. However, it is possible that some of our findings may have been different had the parents been asked to assist their children. Some of our measures were translated into Arabic for this study, and not previously validated in this sample; however, the Cronbach alpha scores indicated their suitability. Finally, we have not assessed pubertal status of the children although this variable may affect their psychological functioning and recommend that this should be done in follow-up research.

Overall, our findings indicate that children with Type 1 diabetes, and their parents, could benefit from targeted psychosocial support. Given that a range of potential issues have been identified in a primary age cohort, such support should be offered early to prevent development of more serious problems later on.

## Supporting information

S1 File (DOCX)
