# Supplementary material for: Exploring the determinants of mental health, wellbeing, and lifestyle in 8–11 year old children with type 1 diabetes and their healthy counterparts in Kuwait
Source: PLoS One. 2022 Dec 12;17(12):e0272948. doi: 10.1371/journal.pone.0272948 (PMC9744296; doi:10.1371/journal.pone.0272948)
Supplement: S1 File — (DOCX) [file pone.0272948.s001.docx]

# **Supporting Information**

**Description of Measures**

***Direct Measures***

HbA1c is the standard medical measure of average blood sugar concentration over the period of 8–12 weeks [1]. HbA1c scores, weight in kilograms, and height in centimetres, were taken from children’s records.

***Self-Report Measures Completed by the Child***

*Self-Esteem*

The Coppersmith Self-Esteem Inventory-School Form (CSEI; Coopersmith, 1967) is a questionnaire that consists of 58 items, which measures self-belief in four subscales (general self, social self, home parent, and school academic). Each item is rated on a 2-point scale either “like me “or “unlike me”. This questionnaire has been found to have good internal consistency (*α*=.78) [2].

*Eating*

The Kids Eating Disorder Survey (KEDS) [3] measures eating disorders and attitudes towards weight and body image. The 12-item questionnaire is comprised of three subscales (body dissatisfaction, disordered eating, and binge eating). However, items eight to ten were excluded in this study. The scale range for all items is 0-2 (0 means the behaviour is not present; 1 that is possibly present, and 2 that it is definitely present). It has good internal consistency (*α*=.73) [3].

*Wellbeing*

The WHO-5 Wellbeing Index (WHO-5) [4] was completed by parents and children as a questionnaire on their quality of life regarding health-related habits in the last two weeks. It is rated on a five-point scale (0 = at no time to 5 = all the time; a higher score indicates better wellbeing) and has good internal consistency (*α*=.89) [4].

*Mental Health*

The Revised Child Anxiety and Depression Scale (RCADS) [5] is a questionnaire to measure aspects of child and adolescent depression and anxiety. It consists of 47 items, which divide into six subscales (social phobia, panic disorder, major depression, separation anxiety, generalized anxiety, and obsessive compulsive). Each item is rated on a four-point Likert scale (never, sometimes, often, and always) in which a higher score indicates a higher occurrence of anxiety and depression related behaviours. This questionnaire has good internal consistency rating (*α*=.76–.95) [6].

*Coping Behaviour*

The Coping Questionnaire for Children and Adolescents (CODI) [7] consists of 29 items, divided into six subscales (acceptance, avoidance, cognitive palliative, distance, emotional reaction, and wishful thinking). Each item is rated on a five-point Likert scale (1 = never, 5 = always) in which a higher score indicates more use of negative coping strategies. The measure has good internal consistency rating (*α*=.69–.83) [7].

***Measures Completed by Parents Regarding Children***

*Mental Health*

The Child Behavior Checklist (CBCL) [8] is a questionnaire by which parents report their child’s behaviour regarding four subscales (depressive problems, anxiety problems, anxious depressed, and withdrawn depressed). It consists of 29 items rated on a three-point Likert scale, (0 = not true to 2 = very true); a higher score indicates an increase in severity for anxiety and/or depression. The measure has been found to have good internal consistency (*α*=.78–.84) [8]**.**

The Strengths and Difficulties Questionnaire (SDQ) [9] was used to measure child’s emotional and behavioural difficulties. It contained 25 items rated on a three-point Likert Scale (0 = not true to 2 = certainly true); a higher score indicates a clinical occurrence of difficulties. The measure consists of six subscales (emotion symptoms, conduct problems, hyperactivity, peer problems, difficulty global score, and prosocial) and has good internal consistency (*α*=.70–.84) [9].

*Sleep Quality*

The Child’s Sleep Habits Questionnaire behaviour (CSHQ-A) [10] measures child sleep behaviours through four subscales (bedtime, sleep behaviour, waking during the night, and morning wake up), and is comprised of 22 items, each rated on a five-point Likert scale (always to never), whereby a high score means the child experiences more sleep disturbances. It has good internal consistency (*α*=.78–.93) [10].

*Lifestyle*

The Lifestyle Behaviour Checklist (LBCL) [11] focuses on the child’s eating, weight, and activities. It is comprised of two subscales; behaviour associated with food (whining, arguing about, and refusing food) and child’s physical and social activity. It has 26 items, overall, rated on a seven-point Likert scale (1 = not at all to 7 = very much), with higher scores on each subscale indicating difficulty in this area. The questionnaire has good internal consistency (*α*=.90) [11].

*Diet*

The Children’s Dietary Questionnaire (CDQ) [12] contains 28 items which measure food intake over the past seven days or 24 hours. These items create five subscales (fruit and vegetables, sweetened beverages, water, fat from dairy, and non-core food which means high fat, salt, or sugar food). Higher scores on all subscales, except fruit and vegetables, suggest unhealthy dietary intake. Overall, this questionnaire has acceptable internal consistency (as subscale scores range from *α*=.51 to .90) [12].

*Activity*

The Physical Activity Questionnaire for Children (C-PAQ) [13] examines how frequently certain activities take place during various times of the week such as spare time, school time, and the weekend, leading to an estimate of a child’s physical activity level. Higher scores suggest higher levels of physical activity at each time point. This questionnaire has good internal consistency (α=.4) [13].

***Parental Self-Report Measures***

*Parental Shame*

The Other as a Shamer (OaS) [14] scale is a questionnaire consisting of 18 items with a five-point Likert scale (0 = never to 4 = always), whereby a higher score suggests parents subjectively experience more shame from external sources. It has a good internal consistency (*α*= .92) [14].

*Parental Coping Behaviour*

The Coping Health Inventory for Parents (CHIP) [15] consists of 45 items, rated on a three-point Likert scale (0 = not helpful to 3 = extremely helpful). A higher score overall suggests that parents use more positive coping strategies. It examines how parents appraise the behavioural techniques they use to manage family life with a child who has a chronic illness. It consists of three subscales Coping 1 (cooperation and an optimistic definition of the situation, family integration), Coping 2 (self-esteem and psychological stability, social support), and Coping 3 (understanding the health care situation through communication with other parents and consultation with the health care-team). It has good internal consistency (*α*=.79) [15].

*Parental Fear of Hypoglycaemia*

Hypoglycaemia Fear Survey (HFS-P) [16] contains 27 items measured on a five-Point Likert scale (1 = never, 5 = very often); a higher score indicates the parents experience more fear regarding hypoglycaemia in their children. The scale consists of two subscales, behaviour and worry, and has good internal consistency (*α*=.89) [16].

*Parental Mental Health*

Depression Anxiety Stress Scales **(**DASS-21) [17] is a questionnaire which measures depression, anxiety, and stress as subscales; it has 21 items which use a four-point Likert scale (0 = did not apply to me at all to 3 = applied to me very much, or most of the time). In addition, a high overall score indicates the occurrence of a clinical condition. This questionnaire has good internal consistency (Stress *α*=.70; depression *α*=.72, and anxiety *α*=.77) [18].

*Parenting Behaviour*

The Parenting Scale [19] contains three subscales (laxness, over reactivity, and verbosity) made from 30 items. Each item consists of two contrasting statements; it is scored on a seven-point Likert scale, where a score of 1 suggests the parent’s behaviour is closest to left-hand statement and a score of 7 suggests the right-hand statement is most fitting; a high score indicates dysfunctional parenting practices. It has good internal consistency (α=.82) [19].

*Parental Child Feeding Behaviour*

The Child Feeding Questionnaire (CFQ) [20] has 31 items, which create seven subscales (responsibility, parental weight, child weight, concern about child weight, pressure to eat, monitoring, and restriction). Three subscales relate to parenting practices and attitudes around feeding, and four relate to parental beliefs about obesity in themselves and their child. Moreover, each item is rated on a five-point Likert scale (1 = the least to 5 = the most). The questionnaire has good internal consistency (above *α*=.70) [20]

**References**

**1**. Little RR, Sacks DB. HbA1c: how do we measure it and what does it mean? Current Opinion in Endocrinology, Diabetes and Obesity. 2009 Apr 1;16(2):113-8.

**2**. Lane GG, White AE, Henson RK. Expanding reliability generalization methods with KR-21 estimates an RG study of the Coopersmith Self-Esteem Inventory. Educational and psychological measurement. 2002 Aug;62(4):685-711.

**3**. Childress AC, Brewerton TD, Hodges EL, Jarrell MP. The Kids' Eating Disorders Survey (KEDS): a study of middle school students. Journal of the American Academy of Child & Adolescent Psychiatry. 1993 Jul 1;32(4):843-50.

**4**. World Health Organization. Wellbeing measures in primary health care: the DepCare project: report on a WHO meeting. Stockholm, Sweden. 1998 Feb 12:12-3.

**5**. Chorpita BF, Yim L, Moffitt C, Umemoto LA, Francis SE. Assessment of symptoms of DSM-IV anxiety and depression in children: A revised child anxiety and depression scale. Behaviour research and therapy. 2000 Aug 1;38(8):835-55.

**6**. Kosters M, Van der Heijden J. From mechanism to virtue: Evaluating Nudge theory. Evaluation. 2015 Jul;21(3):276-91.

**7**. Petersen C, Schmidt S, Bullinger M. Brief report: development and pilot testing of a coping questionnaire for children and adolescents with chronic health conditions. Journal of Pediatric Psychology. 2004 Dec 1;29(8):635-40.

**8.** Achenbach TM, Rescorla LA. Manual for the ASEBA school-age forms & profiles: an integrated system of multi-informant assessment Burlington, VT: University of Vermont. Research Center for Children, Youth, & Families. 2001;1617.

**9.** Goodman R. The Strengths and Difficulties Questionnaire: a research note. Journal of child psychology and psychiatry. 1997 Jul;38(5):581-6.

**10**. Owens JA, Spirito A, McGuinn M. The Children's Sleep Habits Questionnaire (CSHQ): psychometric properties of a survey instrument for school-aged children. Sleep-New York-. 2000 Dec 15;23(8):1043-52.

**11**. West F, Sanders MR. The Lifestyle Behaviour Checklist: A measure of weight‐related problem behaviour in obese children. International journal of pediatric obesity. 2009 Dec;4(4):266-73.

**12**. Magarey A, Golley R, Spurrier N, Goodwin E, Ong F. Reliability and validity of the Children's Dietary Questionnaire; a new tool to measure children's dietary patterns. International Journal of Pediatric Obesity. 2009 Dec;4(4):257-65.

**13**. Anderson YC, Wynter LE, Grant CC, Stewart JM, Cave TL, Wild CE, Derraik JG, Cutfield WS, Hofman PL. Physical activity is low in obese New Zealand children and adolescents. Scientific reports. 2017 Feb 3;7(1):1-7.

**14**. Allan S, Gilbert P, Goss K. An exploration of shame measures—II: psychopathology. Personality and Individual differences. 1994 Nov 1;17(5):719-22.

**15**. McCubbin HI, McCubbin MA, Nevin R, Cauble E. Coping health inventory for parents (CHIP). Family assessment: Resiliency, coping, and adaptation-Inventories for research and practice. 1981:407-53.

**16.** Cox DJ, Irvine A, Gonder-Frederick L, Nowacek G, Butterfield J. Fear of hypoglycemia: quantification, validation, and utilization. Diabetes care. 1987 Sep 1;10(5):617-21.

**17**. Lovibond PF, Lovibond SH. The structure of negative emotional states: Comparison of the Depression Anxiety Stress Scales (DASS) with the Beck Depression and Anxiety Inventories. Behaviour research and therapy. 1995 Mar 1;33(3):335-43.

**18.** Tran TD, Tran T, Fisher J. Validation of the depression anxiety stress scales (DASS) 21 as a screening instrument for depression and anxiety in a rural community-based cohort of northern Vietnamese women. BMC psychiatry. 2013 Dec;13(1):1-7.

**19**. Arnold DS, O'leary SG, Wolff LS, Acker MM. The Parenting Scale: a measure of dysfunctional parenting in discipline situations. Psychological assessment. 1993 Jun;5(2):137.

**20.** Birch LL, Fisher JO, Grimm-Thomas K, Markey CN, Sawyer R, Johnson SL. Confirmatory factor analysis of the Child Feeding Questionnaire: a measure of parental attitudes, beliefs and practices about child feeding and obesity proneness. Appetite. 2001 Jun 1;36(3):201-10.

**Cronbach Alphas for all Measures**

| Measure | Alpha at baseline |
| --- | --- |
| *KEDS* | .76-.95 |
| *Coping* | .69-.83 |
| *Coppersmith Self-Esteem* | .78 |
| *RCADS* | .86 |
| *Wellbeing* | .89 |
| *Parenting Scale* | .82 |
| *Child Feeding Questionnaire* | .70 |
| Shame Total | .92 |
| *DASS-21* | .77 |
| *Parent Wellbeing* | .77 |
| *Coping* | .79 |
| *Sleep Habits* | .78-.93 |
| *Fear of Hypoglycemia* | .89 |
| *Lifestyle Behaviour Checklist* | .90 |
| *CBCL* | .74-.84 |
| *SDQ* | .50 |
| *Children’s Dietary Questionnaire* | .51-.90 |
| *Children’s Physical Activity Questionnaire* | .40 |

**Tables of Descriptive Statistics for Diabetes Group and Control Group**

Descriptive Statistics for Child Self-Completed Measures (Diabetes Group)

| *Measure* | *N* | Min | Max | *M* | *SD* | Skew | Kurtosis |
| --- | --- | --- | --- | --- | --- | --- | --- |
| Level of Blood Sugar (HbA1c) | 100 | 5.50 | 13.00 | 8.64 | 1.23 | 0.39 | 1.17 |
| Child Weight in kg | 100 | 20.00 | 106.00 | 40.93 | 14.7 | 2.40 | 7.49 |
| Child Height in cm | 100 | 125.00 | 164.00 | 141.08 | 9.71 | 0.51 | -0.54 |
| BMI percentiles | 100 | 1.00 | 99.00 | 75.71 | 21.00 | -1.15 | 1.15 |
| Child Wellbeing | 100 | 2.00 | 25.00 | 17.59 | 4.54 | -0.42 | 0.14 |
| *KEDS* |  |  |  |  |  |  |  |
| Eating Disorders | 100 | 0.00 | 9.00 | 4.47 | 2.59 | -0.42 | -1.17 |
| Binge Eating | 100 | 0.00 | 4.00 | 0.46 | 0.78 | 1.93 | 4.12 |
| Body Dissatisfaction | 100 | -1.00 | 5.00 | 1.38 | 1.30 | 0.25 | -0.63 |
| *Coping* |  |  |  |  |  |  |  |
| Acceptance | 100 | 6.00 | 30.00 | 18.41 | 6.51 | 0.00 | -0.67 |
| Avoidance | 100 | 4.00 | 20.00 | 11.75 | 3.66 | 0.22 | -0.24 |
| Cognitive-Palliative | 100 | 9.00 | 25.00 | 18.21 | 3.18 | -0.21 | 0.60 |
| Distance | 100 | 4.00 | 20.00 | 10.24 | 3.69 | 0.00 | -0.52 |
| Emotional Reaction | 100 | 7.00 | 30.00 | 21.20 | 4.26 | -0.57 | 0.53 |
| Wishful Thinking | 100 | 3.00 | 15.00 | 10.99 | 2.98 | -0.28 | -0.64 |
| *Coppersmith Self-Esteem* |  |  |  |  |  |  |  |
| General Self | 100 | 5.00 | 25.00 | 12.20 | 3.26 | 0.36 | 1.46 |
| Social Self | 100 | 0.00 | 7.00 | 3.43 | 1.72 | 0.17 | -0.64 |
| Home Parents | 100 | 0.00 | 7.00 | 3.08 | 1.43 | 0.59 | 0.41 |
| School Academic | 100 | 0.00 | 7.00 | 3.21 | 1.59 | 0.21 | -0.58 |
| Total Score | 100 | 4.50 | 22.00 | 10.96 | 2.83 | 0.43 | 1.15 |
| *RCADS* |  |  |  |  |  |  |  |
| Social Phobia | 100 | 27.00 | 64.00 | 42.30 | 7.05 | 0.37 | 0.83 |
| Panic Disorder | 100 | 36.00 | 82.00 | 56.25 | 8.84 | -0.01 | -0.02 |
| Major Depression | 100 | 32.00 | 83.00 | 52.65 | 9.34 | 0.43 | 0.40 |
| Separation Anxiety | 100 | 36.00 | 77.00 | 55.56 | 8.28 | 0.06 | -0.35 |
| Generalized Anxiety | 100 | 29.00 | 64.00 | 44.38 | 7.46 | 0.26 | -0.10 |
| Obsessive Compulsive | 100 | 5.00 | 72.00 | 48.72 | 9.59 | -0.78 | 3.74 |

Descriptive Statistics for Parent-Completed Self-Report Measures (Diabetes Group)

| *Measure* | *N* | Min | Max | *M* | *SD* | Skew | Kurtosis |
| --- | --- | --- | --- | --- | --- | --- | --- |
| *Parenting Scale* |  |  |  |  |  |  |  |
| Laxness | 100 | 1.91 | 6.55 | 4.34 | 0.78 | -0.04 | 1.45 |
| Over reactivity | 100 | 1.60 | 6.30 | 4.14 | 0.84 | -0.16 | 0.37 |
| Verbosity | 100 | 1.00 | 6.57 | 4.16 | 1.06 | -0.20 | 0.03 |
| Parenting Scale Sum | 100 | 2.03 | 6.30 | 4.28 | 0.67 | -0.15 | 2.38 |
| *Child Feeding Questionnaire* |  |  |  |  |  |  |  |
| Responsibility | 100 | 1.00 | 5.00 | 4.05 | 0.86 | -1.43 | 2.85 |
| Parental Weight | 100 | 1.00 | 4.00 | 3.06 | 0.57 | -0.82 | 1.00 |
| Child Weight | 100 | 1.33 | 4.00 | 2.91 | 0.59 | -0.28 | -0.45 |
| Concern About Child Weight | 100 | 1.00 | 5.00 | 3.35 | 0.97 | -0.21 | -0.53 |
| Restriction | 100 | 2.13 | 5.00 | 3.79 | 0.72 | -0.32 | -0.49 |
| Pressure to eat | 100 | 1.00 | 5.00 | 3.19 | 1.00 | -0.05 | -1.20 |
| Monitoring | 100 | 2.00 | 5.00 | 3.71 | 0.84 | -0.30 | -0.56 |
| Shame Total | 100 | 31.00 | 72.00 | 56.66 | 9.37 | -0.43 | -0.47 |
| *DASS-21* |  |  |  |  |  |  |  |
| Stress | 100 | 0.00 | 17.00 | 7.30 | 3.65 | 0.59 | -0.00 |
| Anxiety | 100 | 0.00 | 16.00 | 6.56 | 3.78 | 0.35 | -0.38 |
| Depression | 100 | 0.00 | 19.00 | 5.21 | 3.55 | 0.87 | 1.41 |
| *Parent Wellbeing* | 100 | 2.00 | 25.00 | 15.95 | 5.55 | -0.33 | -0.06 |
| *Coping* |  |  |  |  |  |  |  |
| Subscale 1 | 100 | 26.00 | 56.00 | 42.58 | 6.87 | -0.20 | -0.57 |
| Subscale 2 | 100 | 23.00 | 54.00 | 39.79 | 6.37 | -0.64 | 0.23 |
| Subscale 3 | 100 | 8.00 | 24.00 | 17.53 | 3.09 | -0.09 | -0.03 |

Descriptive Statistics for Parent-Completed Child Measures (Diabetes Group)

| *Measure* | *N* | Min | Max | *M* | *SD* | Skew | Kurtosis |
| --- | --- | --- | --- | --- | --- | --- | --- |
| *Sleep Habits* |  |  |  |  |  |  |  |
| Sleep Bedtime | 100 | 6.00 | 25.00 | 14.62 | 4.16 | 0.28 | -0.30 |
| Sleep Behaviour | 100 | 4.00 | 22.00 | 9.44 | 3.49 | 0.91 | 0.84 |
| Waking during the night | 100 | 0.00 | 8.00 | 3.77 | 1.36 | -0.17 | 1.39 |
| Morning wake up | 100 | 0.00 | 16.00 | 7.64 | 2.99 | 0.07 | 0.21 |
| *Fear of Hypoglycaemia* |  |  |  |  |  |  |  |
| Behaviour Scale | 100 | 1.10 | 5.00 | 3.82 | 0.78 | -1.21 | 1.92 |
| Worry Scale | 100 | 1.06 | 4.25 | 2.87 | 0.68 | -0.44 | 0.00 |
| *Life style Behaviour Checklist* |  |  |  |  |  |  |  |
| Food | 100 | 4.00 | 45.00 | 20.98 | 8.26 | 0.64 | 0.52 |
| Physical Activity and Situation | 100 | 0.00 | 45.00 | 12.08 | 7.34 | 1.20 | 3.35 |
| *CBCL* |  |  |  |  |  |  |  |
| Depressive Problem | 100 | 0.00 | 15.00 | 5.15 | 2.98 | 0.65 | 0.44 |
| Anxiety Problems | 100 | 0.00 | 10.00 | 4.21 | 1.96 | 0.46 | -0.00 |
| Anxious ‎/ Depressed | 100 | 0.00 | 12.00 | 5.88 | 2.66 | 0.35 | -0.12 |
| Withdrawn ‎/ Depressed | 100 | 0.00 | 10.00 | 3.41 | 2.53 | 0.75 | 0.02 |

| *SDQ* |  |  |  |  |  |  |  |
| --- | --- | --- | --- | --- | --- | --- | --- |
| Emotional Symptoms | 100 | 0.00 | 8.00 | 2.73 | 1.72 | 0.74 | 0.73 |
| Conduct Problem | 100 | 1.00 | 6.00 | 2.67 | 1.13 | 0.38 | -0.04 |
| Hyperactivity | 100 | 0.00 | 5.00 | 2.11 | 1.41 | 0.37 | -0.65 |
| Peer problem | 100 | 0.00 | 7.00 | 3.63 | 1.27 | -0.40 | 0.37 |
| Difficulties Global Score | 100 | 2.00 | 20.00 | 11.14 | 3.57 | 0.20 | 0.31 |
| Prosocial | 100 | 0.00 | 10.00 | 3.68 | 2.17 | 0.60 | 0.17 |
| *Children’s Dietary Questionnaire* |  |  |  |  |  |  |  |
| Fruits eaten in the last 7 days | 100 | 0.00 | 19.00 | 6.59 | 3.86 | 0.88 | 1.13 |
| Fruit last 24 hours | 100 | 0.00 | 10.00 | 2.06 | 1.52 | 1.66 | 6.44 |
| Fruit last week | 100 | 0.00 | 6.00 | 2.97 | 1.41 | -0.18 | -0.65 |
| Veg eaten in the last 7 days | 100 | 0.00 | 14.00 | 5.47 | 3.08 | 0.34 | 0.07 |
| Veg in evening meal in the last 24 hours | 100 | 0.00 | 5.00 | 1.00 | 0.88 | 1.68 | 4.68 |
| Veg last 24 hours | 100 | 0.00 | 5.00 | 0.85 | 0.88 | 1.65 | 5.09 |
| *Continued* |  |  |  |  |  |  |  |
| *Measure* | *N* | Min | Max | *M* | *SD* | Skew | Kurtosis |
| Veg last week | 100 | 0.00 | 6.00 | 2.34 | 1.43 | 0.61 | -0.34 |
| Diary - last 24 hours | 100 | 0.00 | 17.00 | 6.29 | 3.31 | 0.90 | 1.48 |
| Diary Reduced - last 24 hours | 100 | 0.00 | 7.00 | 1.87 | 1.46 | 1.00 | 1.32 |
| Non-core foods past 7 days | 100 | 6.00 | 53.00 | 22.43 | 8.51 | 1.08 | 1.99 |
| Sweetened beverage last 24 hours | 100 | 0.00 | 5.00 | 1.07 | 1.04 | 1.15 | 1.60 |
| Water last 24 hours | 100 | 1.00 | 5.00 | 4.25 | 0.85 | -1.78 | 4.62 |
| Fruit eaten average daily portion | 100 | 0.00 | 4.00 | 1.66 | 0.72 | 0.41 | 0.79 |
| Veg eaten average daily portion | 100 | 0.00 | 3.57 | 1.38 | 0.60 | 0.28 | 0.98 |
| Non-core foods average daily portion | 100 | 0.86 | 7.57 | 3.20 | 1.21 | 1.08 | 1.99 |
| *Children’s Physical Activity Questionnaire* |  |  |  |  |  |  |  |
| Physical activity frequency weekdays | 100 | 0.00 | 6.00 | 2.08 | 1.91 | 0.35 | -0.97 |
| Physical activity total time in mints weekdays | 100 | 0.00 | 120.00 | 36.71 | 35.08 | 0.54 | -0.57 |
| Physical activity weekend frequency | 100 | 0.00 | 0.00 | 0.00 | 0.00 |  |  |
| Physical activity weekend total time in mints | 100 | 0.00 | 0.00 | 0.00 | 0.00 |  |  |
| Leisure activity weekday frequency | 100 | 0.00 | 3.00 | 0.05 | 0.35 | 7.37 | 55.26 |
| Leisure activity weekday time in mints | 100 | 0.00 | 50.00 | 0.95 | 6.69 | 6.99 | 48.00 |
| School active travel | 100 | 0.00 | 0.00 | 0.00 | 0.00 |  |  |
| School activity total time in minutes | 100 | 0.00 | 45.00 | 0.45 | 4.50 | 10.00 | 100.00 |
| Sedentary behaviours total Freq | 100 | 0.00 | 3.00 | 1.45 | 0.62 | 0.06 | -0.22 |
| Sedentary behaviours total Freq | 100 | 0.00 | 3.00 | 1.45 | 0.62 | 0.06 | -0.22 |
| Sedentary behaviours total time in minutes weekdays | 100 | 0.00 | 500.00 | 194.00 | 119.76 | 0.42 | -0.30 |

| Sedentary behaviours total time in minutes weekend | 100 | 0.00 | 1000.00 | 403.10 | 241.46 | 0.50 | -0.49 |
| --- | --- | --- | --- | --- | --- | --- | --- |

Descriptive Statistics for Child Self-Completed Measures (Control Group)

| Measure | *N* | Min | Max | *M* | *SD* | Skew | Kurtosis |
| --- | --- | --- | --- | --- | --- | --- | --- |
| Child Weight in kg | 100 | 20 | 55 | 34.00 | 6.54 | 0.41 | 0.19 |
| Child Height in cm | 100 | 122 | 150 | 133.50 | 5.97 | 0.28 | -0.29 |
| BMI Percentile | 100 | 1.00 | 98.00 | 74.21 | 25.20 | -1.46 | 1.31 |
| *Coppersmith Self-Esteem* |  |  |  |  |  |  |  |
| General Self | 100 | 3.00 | 22.00 | 13.55 | 3.64 | -0.11 | 0.24 |
| Social Self | 100 | 1.00 | 7.00 | 4.01 | 1.47 | 0.06 | -0.31 |
| Home Parents | 100 | 0.00 | 8.00 | 3.91 | 1.63 | -0.00 | -0.31 |
| School Academic | 100 | 0.00 | 7.00 | 4.16 | 1.62 | -0.23 | -0.47 |
| Self-Esteem total | 100 | 2.50 | 19.00 | 12.81 | 3.19 | -0.38 | 0.24 |
| *KEDS* |  |  |  |  |  |  |  |
| Eating Disorder | 100 | 0.00 | 10.00 | 5.58 | 1.97 | -0.89 | 0.12 |
| Binge Eating | 100 | 0.00 | 3.00 | 0.77 | 1.02 | 1.05 | -0.19 |
| Body Dissatisfaction | 100 | -1.00 | 5.00 | 1.54 | 1.50 | 0.35 | -0.90 |
| *Child Wellbeing* | 100 | 10.00 | 25.00 | 21.15 | 3.53 | -0.87 | 0.33 |
| *RCADS* |  |  |  |  |  |  |  |
| Social Phobia | 100 | 27.00 | 55.00 | 35.61 | 5.40 | 0.85 | 1.38 |
| Panic Disorder | 100 | 3.00 | 61.00 | 45.56 | 7.20 | -1.88 | 11.08 |
| Major Depression | 100 | 31.00 | 62.00 | 42.19 | 6.66 | 0.59 | 0.22 |
| Separation Anxiety | 100 | 31.00 | 64.00 | 44.89 | 6.09 | 0.30 | 0.39 |
| Generalized Anxiety | 100 | 28.00 | 52.00 | 36.71 | 5.40 | 0.80 | 0.21 |
| Obsessive Compulsive | 100 | 28.00 | 68.00 | 38.07 | 6.43 | 1.18 | 3.81 |

Descriptive Statistics for Parent-Completed Self-Report Measures (Control Group)

| Measure | *N* | Min | Max | *M* | *SD* | Skew | Kurtosis |
| --- | --- | --- | --- | --- | --- | --- | --- |
| *Shame* *sum* | 100 | 24.00 | 72.00 | 54.95 | 11.79 | -0.74 | -0.06 |
| *Child Feeding Questionnaire* |  |  |  |  |  |  |  |
| Perceived Responsibility | 100 | 1.00 | 5.00 | 3.71 | 0.83 | -0.96 | 1.51 |
| Parent Weight | 100 | 1.00 | 4.00 | 2.90 | 0.61 | -1.03 | 1.07 |
| Child Weight | 100 | 1.00 | 4.00 | 2.89 | 0.49 | -1.30 | 3.64 |
| Concern Child Weight | 100 | 1.00 | 5.00 | 3.19 | 1.05 | -0.25 | -0.68 |
| Restriction | 100 | 1.63 | 5.00 | 3.77 | 0.76 | -0.53 | -0.20 |
| Pressure to Eat | 100 | 1.50 | 5.00 | 3.36 | 0.81 | -0.07 | -0.37 |
| Monitoring | 100 | 2.00 | 5.00 | 3.46 | 0.74 | 0.32 | -0.31 |
| *Parents Wellbeing* | 100 | 0.00 | 25.00 | 17.05 | 4.90 | -0.65 | 0.99 |
| *DASS-21* |  |  |  |  |  |  |  |
| Depression | 100 | 0.00 | 17.00 | 5.91 | 4.17 | 0.47 | -0.79 |
| Anxiety | 100 | 0.00 | 19.00 | 6.25 | 4.23 | 0.44 | -0.29 |
| Stress | 100 | 0.00 | 17.00 | 7.70 | 4.18 | 0.26 | -0.52 |
| *Parenting Scale* |  |  |  |  |  |  |  |
| Laxness | 100 | 2.64 | 7.00 | 4.60 | 0.87 | 0.27 | 0.37 |
| Over reactivity | 100 | 2.70 | 7.00 | 4.28 | 0.83 | 0.63 | 1.05 |
| Verbosity | 100 | 2.29 | 7.00 | 4.59 | 0.94 | 0.22 | 0.41 |
| Parenting Style Sum | 100 | 2.00 | 7.00 | 4.80 | 1.12 | -0.14 | -0.18 |

Descriptive Statistics for Parent-Completed Child Measures (Control Group)

| Measure | *N* | Min | Max | *M* | *SD* | Skew | Kurtosis |
| --- | --- | --- | --- | --- | --- | --- | --- |
| *Sleep Habits* |  |  |  |  |  |  |  |
| Sleep Bedtime | 100 | 0.00 | 32.00 | 17.60 | 6.90 | 0.12 | -0.19 |
| Sleep behaviour | 100 | 0.00 | 28.00 | 9.22 | 4.90 | 0.68 | 1.04 |
| Waking during night | 100 | 0.00 | 8.00 | 1.88 | 1.38 | 0.82 | 2.64 |
| Morning wake up | 100 | 0.00 | 13.00 | 2.84 | 2.78 | 0.81 | 0.68 |
| *CBCL* |  |  |  |  |  |  |  |
| Depressive Problem | 100 | 0.00 | 12.00 | 3.02 | 2.22 | 1.51 | 3.64 |
| Anxiety Problem | 100 | 0.00 | 10.00 | 2.65 | 2.08 | 0.84 | 0.60 |
| Anxious Depressed | 100 | 0.00 | 15.00 | 3.78 | 2.85 | 1.02 | 1.90 |
| Withdraw Depressed | 100 | 0.00 | 7.00 | 1.61 | 1.54 | 0.94 | 0.63 |
| *SDQ* |  |  |  |  |  |  |  |
| Emotional Symptoms | 100 | 0.00 | 6.00 | 1.77 | 1.39 | 0.73 | 0.08 |
| Conduct Problem | 100 | 0.000 | 7.00 | 2.56 | 1.23 | 0.79 | 1.55 |
| Hyperactivity | 100 | 3.00 | 9.00 | 5.28 | 1.37 | 0.34 | -0.44 |
| Peer problem | 100 | 0.00 | 8.00 | 3.87 | 1.36 | -0.15 | 1.27 |
| Difficulties Global Score | 100 | 7.00 | 22.00 | 13.48 | 2.94 | 0.53 | 0.40 |
| Prosocial | 100 | 0.00 | 10.00 | 2.79 | 1.77 | 1.17 | 2.21 |
| *Lifestyle Behaviour Checklist* |  |  |  |  |  |  |  |
| Food | 100 | 2.00 | 45.00 | 15.53 | 8.21 | 0.92 | 1.17 |
| Physical Activity and Situation | 100 | 0.00 | 25.00 | 10.07 | 5.94 | 0.49 | -0.36 |
| *Children’s Dietary Questionnaire* |  |  |  |  |  |  |  |
| Fruits Eaten in the last 7 days | 100 | 0.00 | 18.00 | 4.83 | 2.18 | 2.33 | 13.30 |
| Fruit last 24 hours | 100 | 0.00 | 11.00 | 2.39 | 2.30 | 1.56 | 2.70 |
| Fruit last week | 100 | 0.00 | 6.00 | 2.05 | 1.29 | 0.97 | 0.96 |
| Veg eaten in the last 7 days | 100 | 0.00 | 19.00 | 4.91 | 2.68 | 1.25 | 6.69 |
| Veg in evening meal in the last 24 hours | 100 | 0.00 | 5.00 | 1.21 | 1.23 | 1.17 | 0.95 |
| Veg last 24 hours | 100 | 0.00 | 5.00 | 1.06 | 1.17 | 1.46 | 2.01 |
| Veg last week | 100 | 0.00 | 6.00 | 1.87 | 1.25 | 1.57 | 2.59 |
| Diary-last 24 hours | 100 | 0.00 | 25.00 | 7.57 | 4.33 | 1.08 | 2.27 |
| Diary Reduced-last 24 hours | 100 | 0.00 | 10.00 | 2.45 | 2.28 | 1.09 | 0.68 |
| Non-core foods past 7 days | 100 | 3.00 | 78.00 | 27.46 | 12.52 | 1.081 | 1.81 |
| Sweetened beverage last 24 hours | 100 | 0.00 | 5.00 | 1.99 | 1.35 | 0.74 | -0.2 |

*Continued*

| Measure | *N* | Min | Max | *M* | *SD* | Skew | Kurtosis |
| --- | --- | --- | --- | --- | --- | --- | --- |
| Water last 24 hours | 100 | 1.00 | 5.00 | 3.89 | 1.23 | -0.86 | -0.29 |
| Fruit eaten average daily portion | 100 | 0.00 | 3.57 | 1.32 | 0.56 | 1.12 | 2.44 |
| Veg eaten average daily | 100 | 0.29 | 3.43 | 1.29 | 0.56 | 1.13 | 2.22 |
| Non-core foods average daily portion | 100 | 0.43 | 11.14 | 3.92 | 1.78 | 1.08 | 1.81 |
| *Children’s Physical Activity Questionnaire* |  |  |  |  |  |  |  |
| Physical activity frequency weekdays | 100 | 0.00 | 10.00 | 2.67 | 2.26 | 0.67 | 0.19 |
| Physical activity total time in mins weekdays | 100 | 0.00 | 170.00 | 47.25 | 39.75 | 0.56 | -0.24 |
| Physical activity weekend frequency | 100 | 0.00 | 4.00 | 0.23 | 0.69 | 3.35 | 11.76 |

| Leisure activity weekday frequency | 100 | 0.00 | 8.00 | 0.280 | 1.11 | 4.88 | 26.70 |
| --- | --- | --- | --- | --- | --- | --- | --- |
| Leisure activity weekday time in mins | 100 | 0.00 | 70.00 | 4.00 | 14.19 | 3.53 | 11.46 |
| School active travel | 100 | 0.00 | 0.00 | 0.00 | 0.00 |  |  |
| School activity total time in minutes | 100 | 0.00 | 180.00 | 5.40 | 28.40 | 5.53 | 30.53 |
| Sedentary behaviours total frequency | 100 | 1.00 | 5.00 | 1.50 | 0.65 | 2.05 | 8.01 |
| Sedentary behaviours total time in minutes weekdays | 100 | 0.00 | 1190.00 | 189.60 | 187.17 | 1.94 | 7.34 |
| Sedentary behaviours total time in minutes weekend | 100 | 0.00 | 1100.00 | 361.4 | 216.83 | 1.02 | 1.40 |
